# Positive Microbiological Culture among Patients with Infective Keratitis Visiting the Cornea Unit of a Tertiary Care Centre

**DOI:** 10.31729/jnma.8285

**Published:** 2023-10-31

**Authors:** Leesha Shrestha Joshi, Raju Kaiti, Ranjila Shyangbo, Birkha Bogati

**Affiliations:** 1 Department of Ophthalmology, Nepal Eye Hospital, Tripureshwor, Kathmandu, Nepal; 2 Department of Optometry, Nepal Eye Hospital, Tripureshwor, Kathmandu, Nepal; 3 Department of Optometry, National Academy of Medical Sciences, Mahaboudha, Kathmandu, Nepal

**Keywords:** *corneal ulcer*, *keratitis*, *prevalence*

## Abstract

**Introduction:**

Keratitis is the infection and inflammation of the cornea. Microbial keratitis is a potentially sight-threatening corneal condition. The aim of this study was to find out the prevalence of positive microbiological culture among patients with infective keratitis visiting the Cornea Unit of a tertiary care centre.

**Methods:**

A descriptive cross-sectional study was conducted among patients with a clinical diagnosis of infective keratitis presenting to the Cornea Unit of a tertiary eye care centre from 16 October 2020 to 16 March 2021 after obtaining ethical approval from the Ethical Review Board. After slit-lamp examination, corneal scrapings were performed under aseptic conditions which were subjected to Gram stain, potassium hydroxide preparation and culture for bacterial and fungal pathogens. A convenience sampling method was used. The point estimate was calculated at a 95% Confidence Interval.

**Results:**

Among 428 patients, 337 (78.73%) (73.24-84.33, 95% Confidence Interval) had a positive microbiological culture. A total of 213 (49.76%) of enrolled samples had a prior history of ocular trauma. *Aspergillus* species 68 (20.17%) and *Streptococcus* species 33 (9.79%) were the most common organisms isolated from fungal and bacterial corneal ulcers respectively.

**Conclusions:**

The prevalence of positive microbiological culture among patients with infective keratitis from this study is similar to the pattern reported from similar settings.

## INTRODUCTION

Keratitis is the inflammation of the cornea.^1^ Corneal infections are the second most common cause of monocular blindness in developing countries progressing rapidly, threatening the ocular integrity and producing significant tissue destruction.^2,3^

Microbial keratitis is a potentially vision-threatening corneal infection caused by bacteria, fungi, viruses or parasites.^4^ The epidemiological pattern and causative agents for corneal ulcer varies significantly from country to country and region to region within the same country.^3^ The incidence of corneal ulcers is higher in developing countries than in the US.^2,5^ Keratitis is the most common indication of corneal transplantation in the developing world accounting for almost 50.5%.^6^ Hence determining the etiological factors predisposing to the ulceration and the responsible pathogenic organisms for comprehensive management is important.

The aim of this study was to find out the prevalence of positive microbiological culture among patients with infective keratitis in the Cornea Unit of a tertiary care centre.

## METHODS

A descriptive cross-sectional study was performed among patients with a clinical diagnosis of corneal ulcer in the Cornea Unit of R M Kedia Eye Hospital, Birgunj, Parsa, Nepal from 16 October 2020 to 16 March 2021 after obtaining ethical approval from Ethical Review Board (Reference number: 1037). Patients visiting Cornea unit were included in the study. The corneal ulcer was defined as a break in the corneal epithelium with stromal infiltration associated with signs of inflammation with or without hypopyon. Healing corneal ulcers, viral keratitis, autoimmune conditions like Mooren's ulcers, marginal keratitis, interstitial keratitis, and neurotrophic ulcers were excluded. Convenience sampling method was used. The sample size was calculated using the following formula:


$$\begin{array}{ll} \text{n} & ={\text{Z}}^{2}\times \frac{\text{p}\times \text{q}}{{\text{e}}^{\text{2}}}\\ & =1.96^{2}\times \frac{0.50\times 0.50}{0.05^{2}}\\ & =385 \end{array}$$

Where,

n = minimum required sample size
Z = 1.96 at 95% Confidence Interval (CI)
p = prevalence taken as 50% for maximum sample size
q = 1-p
e = margin of error of, 5%

After adjusting for a 10% non-response rate, the final sample was 423. However, 428 patients were enrolled in this study. A detailed history of ocular trauma and diabetes mellitus was taken. All the patients underwent eye examinations: distant visual acuity and near vision assessment. The best corrected visual acuity was noted for each participant. Then, a slit lamp biomicroscopy examination was performed by an experienced ophthalmologist to evaluate the morphology of the corneal ulcer. The size of the epithelial defect was measured after staining with the fluorescein. The presence or absence of hypopyon was also recorded. To evaluate the patency of the nasolacrimal duct, syringing was performed in each patient.

After a detailed ocular examination, corneal scrapings were performed under aseptic conditions on each ulcer by an ophthalmologist using a flame-sterilised Kimura spatula. Scrapings were performed in the slit lamp after the instillation of 4% lignocaine (lidocaine). The material was obtained from scraping, and the leading edge and the base of each ulcer were inoculated directly onto Blood agar, Chocolate agar and Sabaraud-Dextrose agar (SDA). Material from the corneal scraping was also taken on two separate glass slides for smear: one for Gram stain and the other for microscopic examination in the clinic as a KOH wet mount. All KOH smears were then sent to the laboratory for confirmation. All bacterial cultures were incubated aerobically at 37°C. Cultures on blood agar, and chocolate agar were evaluated at 24 hours and at 48 hours and then discarded if there was no growth. Fungal cultures inoculated onto SDA were incubated at 27°C, examined daily, and discarded after 2 weeks if no growth was present in the culture.

Microbial cultures were considered positive only if the growth of the same organism was demonstrated on two or more solid media, or there was semi-confluent growth at the site of inoculation on one solid medium associated with the identification of the organism of appropriate morphology and staining characteristics on Gram stain or KOH mounted corneal smears. The specific identification of bacterial pathogens was based on microscopic morphology, staining characteristics, and biochemical properties using standard laboratory criteria. Fungi were identified by their colony characteristics on SDA and by their microscopic appearance in lacto phenol cotton blue.

Data were entered in a pre-designed format documenting socio-demographic and clinical parameters and analyzed on IBM SPSS Statistics version 26.0. Point estimate was calculated at at 95% CI.

## RESULTS

Among 428 samples, 337 (78.73%) (73.24-84.33, 95% CI) had a positive culture report on microbiological evaluation. Bacterial and fungal isolates could be recovered in 86 (25.51%) and 155 (45.99%) positive samples respectively (Table 1).

**Table 1:** Micro-organisms isolated from positive cultures (n= 337).

Causative organism		n (%)
Bacterial corneal ulcer	*Streptococcus*	33 (9.79)
	*Staphylococcus*	29 (8.60)
	*Pseudomonas*	16 (4.74)
	*Escheria*	6 (1.78)
	*Moraxella*	2 (0.59)
Fungal corneal ulcer	*Aspergillus*	68 (20.17)
	*Fusarium*	47 (13.94)
	*Candida*	23 (6.82)
	*paecilomyces*	12 (3.56)
	*Scedosporium*	5 (1.48)
Mixed corneal ulcer		72 (21.36)
Unidentified		24 (7.12)

Mixed etiologies were observed in 72 (21.36%) culture smears. The most common causative organism in this study was *Aspergillus* species which was seen in 68 (20.17%) smears, followed by *Fusarium* species 47 (13.94%). *Streptococcus* species was isolated in 33(9.79%) cases which was the most common etiology of bacterial corneal ulcer. Out of 155 (45.99%) patients who were positive for fungal isolates, 132 (39.16%) had a positive history of ocular trauma with vegetative materials. Only 26 (7.71%) patients who tested positive for both fungal and bacterial ulcers, had prior history of any trauma.

Out of 337 enrolled patients who had positive culture 176 (52.25%) were male. About 213 (47.76%) patients had a prior definitive history of ocular trauma, whereas 106 (24.76%) patients had diabetic mellitus.

The mean age of the patients with positive culture was 47.98±17.07 years. Among diabetic subjects, the mean random blood sugar level was 114.91±27.84 mg/dl. The mean age of male was 48.47±15.59 years and that of female was 47.33±13.42 years.

A total of 341 (79.67%) patients had patent nasolacrimal duct while performing syringing, whereas regurgitation was observed in 87 (20.32%) patients.

## DISCUSSION

In this study, 78.73% had a positive culture report on microbiological evaluation which is similar to previous studies conducted in similar settings.^7,8^ In the present study, infective keratitis was seen in a preponderance of males than in females, and this finding is also consistent with previous studies from Nepal, India and Ghana.^9^'^11^

In this study, fungal isolates were seen in the majority of the cases followed by bacterial isolates. This finding could have been attributed to the geographical distribution and occupation of Birjung. Located in southern Terai, people from Birgunj are mostly farmers involved in agriculture and livestock farming, predisposing them to vegetative ocular trauma which is the most common cause of fungal keratitis. Corneal trauma disrupts the protective mechanism of the corneal epithelium, facilitating microbial adhesion and accelerating the penetration and replication of microorganisms. Moreover, about 49.76% of the enrolled patients had a prior history of definitive ocular trauma, which further explains the predominance of fungal keratitis over bacterial. It has been estimated that a history of trauma can be observed in 40-92% of all cases of fungal keratitis.^12^ Besides, 24.76% of patients had diabetes mellitus. Systemic diseases such as diabetic mellitus should also be considered as a predisposing factor in infective keratitis as diabetes not only results in diabetic keratopathies but will slow down the wound healing process.

Among the causative agents in fungal keratitis, the most common causative organism was *Aspergillus* species (20.17%), followed by *Fusarium* species (13.94%). This finding is in agreement with previous Nepalese studies from eastern Nepal and other regions and Indian studies.^1,9,10,13^ However, other studies from Nepal have also reported Fusarium species to be the most common microbiological entity, especially from Western Nepal and some studies have reported *Aspergillus* and *Fusarium* to be found in *Aspergillus* in equal proportion.^14,15^ In this study, among the bacterial isolates the most frequent agent identified was *Streptococcus pneumoniae,* followed by *Staphylococcus aureus,* and *Pseudomonas aeruginosa,* which is in agreement with previous reports of Nepal.^7^

The incidence of infective keratitis in Nepal is among the highest reported in the world, which if left untreated can lead to permanent visual impairment and other significant morbidities corneal scar, corneal perforation, and endophthalmitis. Hence, it is important to recognize the common microbial agent for prompt diagnosis and to achieve an effective therapeutic strategy.

Since it is a single-centred study, the findings can not be generalized to whole population.

## CONCLUSIONS

The prevalence of positive culture among patients of infective keratitis visiting the Cornea Unit was similar to other studies done in similar settings. Fungal isolates were most commonly seen. In infective keratitis, it is important to perform cultural investigations for accurate diagnosis of the involved micro-organisms which will guide through the treatment.
